# Computed Tomography Embolus Texture Analysis as a Prognostic Marker of Acute Pulmonary Embolism

**DOI:** 10.1177/00033197221111862

**Published:** 2022-08-16

**Authors:** Jakob Leonhardi, Nikolaos Bailis, Marianne Lerche, Timm Denecke, Alexey Surov, Hans-Jonas Meyer

**Affiliations:** 1 Department of Diagnostic and Interventional Radiology, 39066 University of Leipzig, Leipzig, Germany; 2 Department of Respiratory Medicine, University Hospital Leipzig, 39066 University of Leipzig, Leipzig, Germany; 3 Department of Radiology and Nuclear Medicine, 9376 Otto von Guericke University, Magdeburg, Germany

**Keywords:** texture analysis, computed tomography, pulmonary embolism

## Abstract

Texture analysis is a quantitative imaging analysis that provides novel biomarkers beyond conventional image reading. Our aim was to use texture analysis of pulmonary emboli derived from thoracic computed tomography for prediction of mortality and prognosis of acute pulmonary embolism (PE). Overall, 216 patients (116 female, 53.7%) were included in the analysis. Texture analysis was calculated on axial slices of the contrast enhanced pulmonary angiography of the proximal embolus. Clinical scores, serological parameters, need for intubation, intensive care unit (ICU) admission and mortality was assessed and correlated with the texture features. In the correlation analysis, there were several associations with mortality in days, the highest for the parameter S(0,5)SumVarnc (*r* = −0.43, *P* < 0.001). Another parameter, S(3,−3)AngScMom correlated with sepsis-related organ failure assessment score (SOFA)-score (*r* = 0.31, *P* < 0.001). Several texture features correlated with venous lactate and glucose levels. In discrimination analysis, there were significant differences in regard to texture features between survivors and non-survivors and between patients with and without the need for ICU admission (*P* = 0.02, respectively). These results highlight the potential clinical benefit of texture features in patients with acute PE as novel imaging biomarkers. Further studies are needed to validate these results.

## Introduction

Acute pulmonary embolism (PE) is life-threatening with 30‐day mortality rates ranging from 0.5% to over 20% depending on symptoms at presentation.^1,2^ However, there are also low-risk clinical courses without severe complications.^3^ That is why, an immediate risk stratification of patients with acute PE at presentation is crucial for patient care.^3^

Computed tomography pulmonary angiography (CTPA) is the diagnostic clinical gold standard for the diagnosis of PE with a reported sensitivity and specificity of up to 100%.^4-6^ It is the first imaging performed in patients suspected of PE, most commonly on hospital admission.^4^ Therefore, risk stratification based upon CTPA is important.^5^ Established imaging signs for severe course are the right ventricle diameter to left ventricle (LV) diameter-ratio, which has the strongest predictive value and contrast media reflux into the inferior vena cava, which is a significant prognostic marker in acute PE.^5,7,8^

Texture analysis is an emergent research field to provide novel quantitative biomarkers derived from radiological images.^9-11^ Various applications of texture analysis have been investigated throughout clinical medicine, predominantly in the field of oncology.^9-11^ Different spatial characteristics of tumors were used for better discrimination purposes, treatment prediction and prognosis stratification.^9-11^ Texture analysis by this approach can provide information from different images, which is beyond the scope of the clinical observation carried out by a radiologist.

Regarding thrombus imaging, a plethora of investigations were performed to better characterize cerebral thrombi in acute ischemic stroke patients.^12,13^ Briefly, direct correlation analyses between histologic characteristics and imaging features were performed on unenhanced CT images and CT angiography.^12^ Noteworthy, imaging could predict thrombus permeability, which could aid in mechanical thrombectomy in cerebral stroke.^13^ That is of clinical importance, as clot composition shows statistically significant associations with treatment outcome.^14,15^

Albeit of these promising reports in ischemic stroke patients, the possible benefit of embolus imaging has not been used in PE. There is no doubt that images analyzed with texture analysis can provide insight into the microstructure of the embolus.

Therefore, the present study investigated whether texture analysis parameters of pulmonary emboli derived from CT images show associations with mortality and clinically relevant factors in patients with acute PE.

### Patients and Methods

#### Patient Acquisition

This retrospective study was approved by the institutional review board (118/19-ck, Ethics Committee, University of Leipzig, Leipzig, Germany).

All patients with acute PE were retrospectively assessed within the time period 2014–2019.

Inclusion criteria

- sufficient CT images with clearly visible pulmonary emboli on admission to the hospital;
- available clinical data regarding clinical signs, serological parameters and follow-up.
- no thrombolysis before and/or during the CT acquisition.

Exclusion artifacts were

- severe image artifacts (ie, due to implants or motion artifacts) as well as any form of treatment;
- missing clinical data/follow-up;
- thrombolysis before CT imaging;
- chronic PE.

Overall, 216 patients (116 females, 53.7%) were included in the analysis. The median age at the time of CT acquisition was 65 years ranging from 17 to 99 years. The patient sample was based on a previous study, which elucidated the associations between clinical parameters, survival, and pulmonary CT obstruction scores in patients with acute PE.^16^

### Clinical Parameters

The following clinical parameters were retrieved on hospital admission:

- relevant clinical comorbidities (active malignant disease, surgery performed within the last 4 weeks, chronic lung disease, chronic heart failure).
- blood pressure (mmHg), heart rate (beats/min), need for intubation, vasopressor, or intensive care unit admission.
- following clinical scores were calculated according to the proposed standards: Wells score,^17^ revised Geneva score,^18^ and simplified Pulmonary Embolism Severity Index (sPESI) score.^19^
- serological parameters: hemoglobin (mmol/L), hematocrit (L/L), platelet count (10^9^/L), fibrinogen (g/L), INR (1), PTT (s), D-dimer level (μg/mL), lactate (venous blood, mmol/L), pH (venous blood), glucose (mmol/L), troponin T (μg/L), and N-terminal natriuretic peptide (BNP, pg/mL).
- Mortality, assessed in days after diagnosis of PE.

A risk stratification of PE was performed according to the American Heart Association (AHA) as follows: low-risk PE, submassive PE and massive PE.^3^

Embolus localization was classified as follows: pulmonary trunk, main pulmonary arteries, lobar and segmental arteries according to the most proximal located embolus.

### Imaging Technique

Computed tomography was performed on admission for every patient without any previous treatment.

Computed tomography pulmonary angiography was performed with a 128-slice CT scanner (Ingenuity 128, Philips, Hamburg, Germany). Intravenous administration of an iodine-based contrast medium (60 mL Imeron 400 MCT, Bracco Imaging Germany GmbH, Konstanz, Germany) at a rate of 4.0 mL/s via a peripheral venous line. Automatic bolus tracking was performed in the pulmonary trunk with a trigger of 100 Hounsfield units (HU). Typical imaging parameters were: 100 kVp; 125 mAs; slice thickness, 1 mm; pitch, 0.9. Computed tomography pulmonary angiography was performed in every case in deep inspiration level.

### Texture Analysis

Computed tomography images were processed with the free available texture analysis software MaZda (version 4.7, available at http://www.eletel.p.lodz.pl/mazda/).^20,21^ A polygonal region of interest (ROI) was placed on the largest, representative slide of the pulmonary embolus on the largest, proximal position. The ROI was drawn clearly within the margin of the embolus with at least 2 mm distance to the surrounding contrast media to address possible artifacts. The measurements were performed in a blinded manner to the clinical results by a resident of radiology with 2 years of general experience.

For each ROI, gray-level (μ) normalization was performed, using the limitation of dynamics to μ ± 3 standard deviations to minimize the influence of contrast and brightness variation, as in similar studies utilizing texture analysis.^22,23^ Table 1 provides a detailed description of the investigated texture features. Last, 279 texture features were retrieved for every patient. To ensure intraobserver reliability, measurements were carried out by the same radiologist for a second time in a blinded manner. Intraclass correlation coefficients (ICC) were then calculated for the area of the measured emboli and for four of the most relevant texture features.

**Table 1.** Overview of the Texture Features in the Present Analysis.

Texture group	Texture feature name	Abbreviation
Gray-level histogram	Mean, variance, skewness, kurtosis, percentiles (1, 10, 50, 90, 99%)	Mean
		Variance
		Skewness
		Kurtosis
		Perc.01%, perc.10%, perc.50%, perc.90%, perc.99%
Co-occurrence matrix	Angular second moment, contrast, correlation, entropy, sum entropy, sum of squares, sum average, sum variance, inverse difference moment, difference entropy, difference variance; (for four directions and 5 interpixel distances (offsets; n = 1–5))	S(2,-2)AngScMom, S(3,-3) AngScMom, S(1,-1) AngScMom, S(5.5) AngScMom, S(0.2) AngScMom
		S(3.0) contrast, S(3.3) contrast, S(2.2) contrast, S(4.4) contrast, S(5.0) contrast, S(0.1) contrast
		S(5.0) correlat, S(5,-5) correlat, S(2,-2) correlat, S(1,-1) correlat, S(0.1) correlat, S(3,-3) correlate
		S(3.0) entropy, S(5,-5) entropy, S(2,-2) entropy, S(4,-4) entropy, S(0.5) entrop, S(3,-3) entropy
		S(1.0) SumEntrp, S(0.2) entropy, S(0.5) SumEntrp, S(4.0) SumEntrp, S(3.0) SumEntrp
		S(3.0) SumOfSqs, S(4.0) SumOfSqs, S(0.5) SumOfSqs, S(5,-5) SumOfSqs
		S(2,-2) SumAverg, S(3,-3) SumAverg, S(0.3) SumAverg, S(0.5) SumAverg, S(0.4) SumAverg
		S(1.1) SumVarnc, S(3.0) SumVarnc, S(5,-5) SumVarnc
		S(0.3) InvDfMom, S(1.0) InvDfMom, S(4,-4) InvDfMom, S(5.0) InvDfMom, S(0.1) InvDfMom
		S(5.5) DifEntrp, S(4.4) DifEntrp, S(1,-1) DifEntrp, S(0.1) DifEntrp, S(2.0) DifEntrp, S(1.0) DifEntrp
		S(3.3) DifVarnc, S(1,-1) DifVarnc, S(2,-2) DifVarnc, S(5,-5) DifVarnc, S(2.0) DifVarnc
Run-length matrix	Run-length non-uniformity, gray-level non-uniformity, long run emphasis, short run emphasis, fraction of image in runs; (for 4 angles)	Vertl_GLevNonU,45dgr_GLevNonU, horzl_GLevNonU, 135dr_GLevNonU, Z_GLevNonU
		Vertl_RLNonUni, 45dgr_RLNonUni,135dr_RLNonUni
		Vertl_LngREmph, 45dgr_LngREmph, horzl_LngREmph, 135dr_LngREmph
		Vertl_ShrtREmp, 45dgr_ShrtREmp, horzl_ShrtREmp, 135dr_ShrtREmp
		Vertl_fraction, 45dgr_fraction, horzl_fraction, 135dr_fraction, Z_fraction
Absolute gradient	Gradient mean, variance, skewness, kurtosis, non-zeros	GrMean
		GrVariance
		GrSkewness
		GrKurtosis
		GrNonZeros
Autoregressive model	Theta 1–4, sigma	Teta1, teta2, teta3, teta4
		Sigma
Wavelet transform	Energies of wavelet transform coefficients in sub-bands LL, LH, HL, HH; (for 3 subsampling factors)	WavEnLL_s-1, WavEnLL_s-2, WavEnLL_s-3, WavEnLL_s-4
		WavEnLH_s-1, WavEnLH_s-2, WavEnLH_s-3
		WavEnHL_s-1, WavEnHL_s-2, WavEnHL_s-3, WavEnHL_s-4
		WavEnHH_s-1, WavEnHH_s-2, WavEnHH_s-3

**Figure 1.**
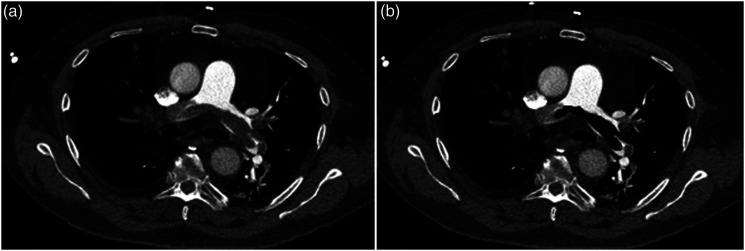
A. Representative case of the patient sample with a central acute pulmonary embolism with saddle thrombus of the truncus pulmonalis. The patient died after 1 day. B. Region of interest (ROI) drawn within the central embolism to measure the texture features. Some smaller emboli were not included into the ROI.

**Figure 2.**
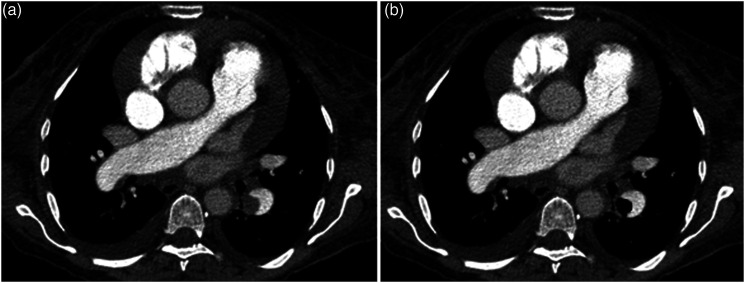
A. Another representative case with a lobar pulmonary embolism in the left lower lobe. One can also appreciate global cardiomegaly, a pleural effusion on the right side as well as a pericardial effusion. The patient survived. B. ROI drawn within the embolism.

Figures 1 and 2 display 2 representative cases of the patient sample for illustration purposes.

### Statistical Analysis

The statistical analysis and graphics creation were performed using GraphPad Prism 5 (GraphPad Software, La Jolla, CA, USA). Collected data were evaluated by means of descriptive statistics (absolute and relative frequencies). Spearman’s correlation coefficient (r) was used to analyze associations between investigated scores after testing for normality distribution. Group differences were calculated with Mann-Whitney test and Fisher exact test, when suitable. ICC was used to calculate intraobserver agreement. Multivariate regression analysis was used to predict ICU admission. Multivariate Cox regression analysis was used to assess mortality outcome. In all instances, a 2-sided *P* < 0.05 was considered statistically significant.

## Results

### Clinical Signs and Scores

Overall, 57 patients (26.4%) died with a median duration of day of 2 (IQR 10), ranging from 1 to 60 days. Furthermore, 172 patients (79.6%) needed ICU admission; 56 patients (25.8%) showed clinical signs of deep venous thrombosis.

Table 2 displays the investigated clinical and serological parameters divided by survivors and non-survivors.

**Table 2.** Overview of the Investigated Demographic and Clinical Parameters. Parameters Marked with * Show Values as Median and Interquartile Range. The Other Parameters Values are Shown as Mean ± Standard Deviation.

Parameter	Non-survivors (n = 57)	Survivors (n = 159)	*P*-value
Age (years)	67.6 ± 16.3	62.9 ± 17.1	0.06
Gender (male, n, %)	32 (54.7)	84 (52.8)	0.67
Systolic blood pressure (mmHg)	105.5 ± 32.9	131.9 ± 22.4	<0.0001
Heart rate (beats/min)	116.3 ± 27.1	102.7 ± 19.3	0.05
Respiratory rate (/min)	23.0 ± 8.7	23.4 ± 7.4	0.95
Body temperature (°C)	36.6 ± 1.2	36.7 ± 0.9	0.79
BMI (kg/m^2^)	29.0 ± 7.7	28.3 ± 5.9	0.83
Troponin T (μg/L)*	74.70 (93.3)	55.29 (77.6)	0.34
D-dimer (μg/ml)*	13.11 (20.9)	7.31 (12.8)	0.19
Venous pH	7.34 ± 0.08	7.37 ± 0.12	<0.0001
Venous lactate (mmol/L)*	3.5 (4.0)	2.2 (1.6)	0.001
Wells score	6.2 ± 1.1	6.1 ± 1.9	0.95
Geneva score	7.3 ± 2.2	8.1 ± 3.7	0.31
sPESI score	3.2 ± 1.1	1.7 ± 0.9	<0.0001
Intubation (n, %)	42 (73.6)	27 (17.1)	<0.0001

Abbreviations: BMI = body mass index, sPESI score = simplified Pulmonary Embolism Severity Index.

Systolic blood pressure, pH, sPESI score and need for intubation were highly significantly different between survivors and non-survivors (each *P* < 0.0001).

As relevant comorbidities, 59 patients (27.2%) had active malignant disease, 51 patients (23.5%) underwent surgery within 4 weeks before the CT scan, 26 patients (11.9%) had chronic lung diseases, and 39 (17.9%) patients had chronic heart failure.

### Localization and Texture Features of Thrombotic Emboli

In 13 patients (6.0%), the embolus was located within the pulmonary trunk. In 97 patients (44.6%), the embolus was located within the main pulmonary arteries. In the other 107 patients (49.4%), the emboli were in the lobar and/or segmental arteries. Table 3 gives an overview of the investigated texture features in accordance with thrombus localization. Most texture features showed statistically significant differences between centrally located (pulmonary trunk and main pulmonary arteries) compared with peripheral located emboli.

**Table 3.** Comparison of the Clinically Relevant Texture Features in Accordance to Thrombus Localization.

Parameter	Pulmonary trunk	Main arteries	Lobar and segmental arteries	*P*-value
S(1,1) Sumvarnc	388.8 ± 25.9	382.9 ± 25.5	369.8 ± 36.9	0.025
S(1,0) InvDfMom	0.30 ± 0.09	0.29 ± 0.06	0.26 ± 0.06	<0.001
S(1,1) SumEntrp	1.88 ± 0.02	1.83 ± 0.08	1.75 ± 0.10	<0.001
S(2,0) SumEntrp	1.87 ± 0.02	1.82 ± 0.08	1.72 ± 0.11	<0.001
S(2,0) DifEntrp	1.16 ± 0.17	1.15 ± 0.10	1.19 ± 0.10	0.091
S(2,2) SumEntrp	1.83 ± 0.04	1.79 ± 0.08	1.68 ± 0.13	<0.001
S(3,0) DifEntrp	1.27 ± 0.15	1.27 ± 0.09	1.27 ± 0.10	0.911
S(3,3) Correlat	0.36 ± 0.30	0.32 ± 0.23	0.16 ± 0.33	0.001
S(3,3) SumEntrp	1.80 ± 0.04	1.76 ± 0.08	1.60 ± 0.24	<0.001
S(4,4) Correlat	0.32 ± 0.29	0.26 ± 0.23	0.06 ± 0.32	<0.001
S(4,-4) InvDfMom	0.11 ± 0.03	0.10 ± 0.03	0.09 ± 0.04	0.019
S(5,5) InvDfMom	0.11 ± 0.03	0.10 ± 0.03	0.08 ± 0.04	<0.001
S(5,0) InvDfMom	0.12 ± 0.04	0.11 ± 0.03	0.10 ± 0.06	0.002
WavEnLL_s-1	17898.10 ± 604.9	17863.71 ± 711.2	18604.57 ± 1473.9	0.001
Horzl_LngREmph	1.52 ± 0.25	1.61 ± 0.38	1.44 ± 0.25	<0.001
Horzl_ShrtREmp	0.91 ± 0.03	0.90 ± 0.04	0.92 ± 0.03	0.001
Horzl_fraction	0.88 ± 0.05	0.86 ± 0.06	0.89 ± 0.05	<0.001
135dr_ShrtREmp	0.94 ± 0.02	0.94 ± 0.03	0.95 ± 0.03	<0.001
135dr_fraction	0.92 ± 0.03	0.92 ± 0.04	0.94 ± 0.03	<0.001

### Intraclass Correlation Coefficient

The calculation of intraclass correlation coefficient for the measured area of the emboli resulted in an ICC of 0.99. For all included texture features, the ICC was over 0.80 showing that agreement was good to excellent. For example, for both S(3,-3)AngScMom and S(0,5)SumVarnc, ICC was 0.83. For S(4,0)InvDfMom, ICC was 0.86 and for S(3,3)Correlat it was 0.88.

### Associations Between Thrombus Texture Features and Outcomes

**Figure 3.**
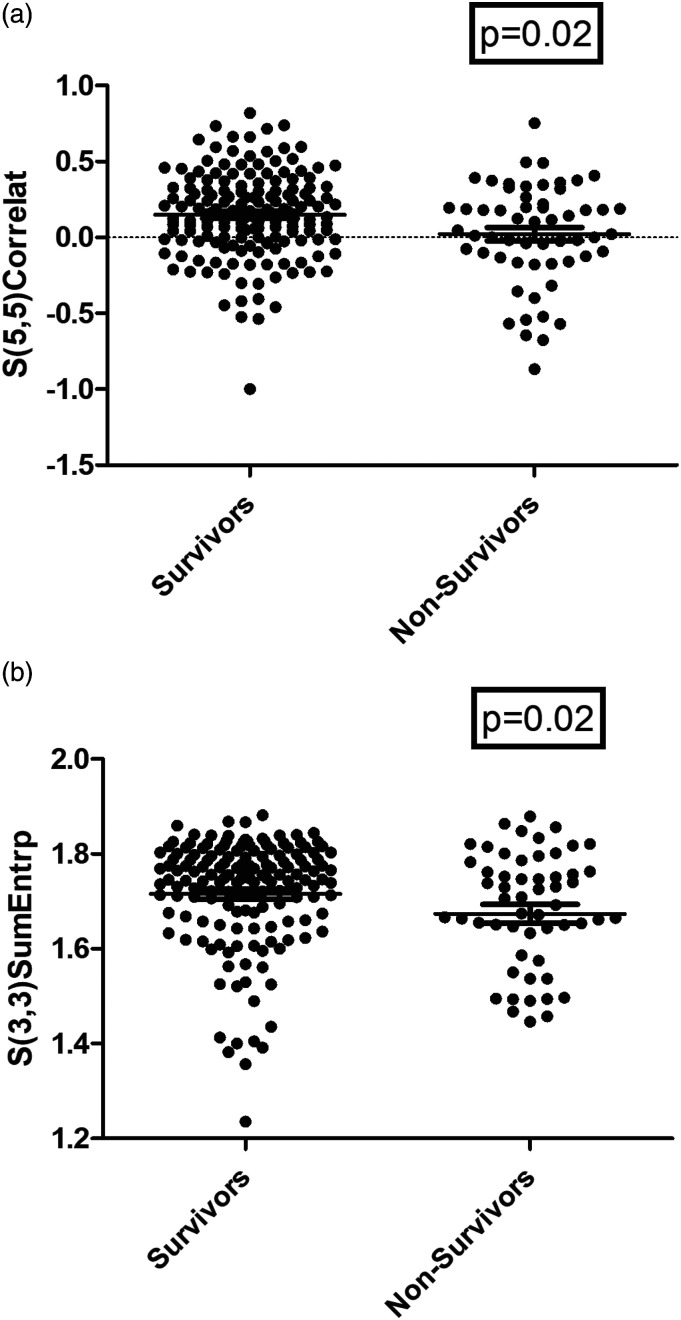
Discrimination between survivors and non-survivors using different texture analysis features. A. Survivors had lower S(5,5)Correlat vs non-survivors, 0.15 ± 0.29 vs 0.02 ± 0.33, respectively (*P* = 0.02). B. Survivors had higher S(3,3)SumEntrp vs non-survivors, 1.71 ± 0.13 1.67 ± 0.15, respectively (*P* = 0.02).

In discrimination analysis, the co-occurrence matrix group of texture features were different between survivors and non-survivors. In detail, S(3,3)Correlat was higher in survivors (0.29 ± 0.27) vs non-survivors (0.16 ± 0.34), *P* = 0.02 (figure 3A). Also S(3,3)SumEntrp was higher in survivors (1.71 ± 0.13) vs non-survivors (1.64 ± 0.27), *P* = 0.015 (figure 3B), (Table 4).

**Table 4.** Comparison of Texture Features in Survivors and Non-Survivors.

Parameter	Non-survivors (n = 57)	Survivors (n = 159)	*P*-value
S(1,1) SumVarnc	370.1 ± 33.00	380.80 ± 30.49	0.021
S(1,1) SumEntrp	1.78 ± 0.10	1.81 ± 0.10	0.024
S(2,0) SumEntrp	1.76 ± 0.11	1.79 ± 0.10	0.029
S(2,2) SumEntrp	1.71 ± 0.13	1.76 ± 0.11	0.018
S(3,3) Correlat	0.16 ± 0.34	0.29 ± 0.27	0.020
S(3,3) SumEntrp	1.64 ± 0.27	1.71 ± 0.13	0.015
S(4,4) Correlat	0.09 ± 0.31	0.21 ± 0.28	0.016
S(4,-4) InvDfMom	0.09 ± 0.03	0.10 ± 0.03	0.028
S(5,5) InvDfMom	0.08 ± 0.04	0.10 ± 0.03	0.017
WavEnLL_s-1	18579.74 ± 1439.92	18025.18 ± 979.90	0.020

Also, several texture analysis parameters were different between the patients with and without the need for ICU admission (Table 5). In multivariate regression analysis, these texture features contributed to prediction model including Wells score, GENEVA score, age and heart rate. An example is shown for the texture feature S(2,0)DifEntrp in Table 6.

**Table 5.** Comparison of the Texture Features in Patients with ICU Admission and Without ICU Admission.

Parameter	ICU admission (n = 172)	No ICU admission (n = 44)	*P*-value
S(1,0) InvDfMom	0.28 ± 0.07	0.26 ± 0.06	0.032
S(2,0) DifEntrp	1.16 ± 0.11	1.20 ± 0.11	0.022
S(3,0) DifEntrp	1.26 ± 0.10	1.30 ± 0.11	0.031
S(5,0) InvDfMom	0.11 ± 0.05	0.10 ± 0.03	0.049
Horzl_LngREmph	1.56 ± 0.36	1.42 ± 0.21	0.024
Horzl_ShrtREmp	0.91 ± 0.04	0.92 ± 0.03	0.007
Horzl_fraction	0.87 ± 0.05	0.89 ± 0.04	0.016
135dr_ShrtREmp	0.94 ± 0.03	0.95 ± 0.02	0.028
135dr_fraction	0.92 ± 0.04	0.94 ± 0.03	0.045

ICU = intensive care unit.

**Table 6.** Multivariate Regression Analysis for ICU Admission.

Parameter	β	95% CI	*P*-value
Wells score	0.88	0.62–1.23	0.44
Geneva score	1.15	0.97–1.37	0.11
Age at embolism	1.01	0.99–1.03	0.40
Heart rate	1.02	1.01–1.04	0.01
S(2,0) DifEntrp	0.02	0.001–0.56	0.02

Abbreviation: CI = Confidence interval, β = regression coefficient.

Overall, 21 patients were classified as massive PE (9.7%), 186 patients were submassive (86.1%) and 9 patients were of low-risk (4.2%).

S(4,0)InvDfMom derived from the co-occurrence matrix group could discriminate between massive vs submassive PE (*P* = 0.02).

In a further analysis, the submassive group was classified with patients with clinical deterioration defined by ICU admission and intubation (n = 54, 29.0%) and patients without clinical deterioration (n = 132, 71.0%).

**Figure 4.**
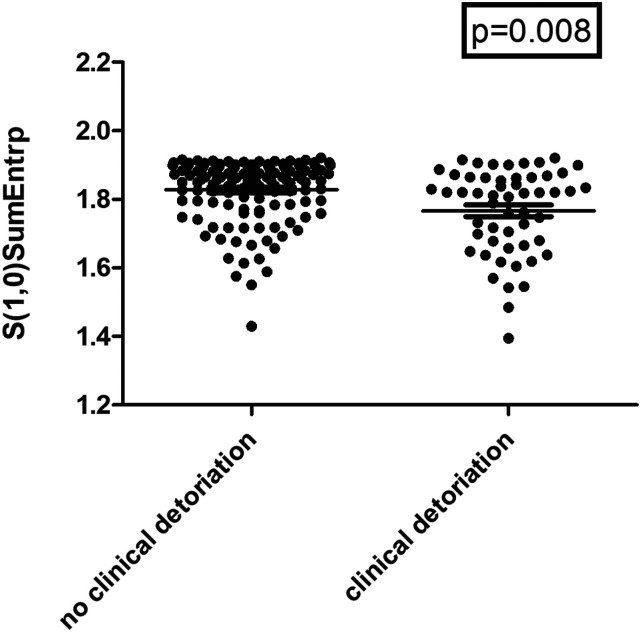
S(1,0)SumEntrp can discriminate patients with clinical deterioration defined by ICU admission and patients without clinical deterioration, 1.83 ± 0.09 vs 1.77 ± 0.12, respectively (*P* = 0.008).

Several texture features were different between these groups. The highest statistically significance achieved S(1,0)SumEntrp (1.83 ± 0.09 vs 1.77 ± 0.12, *P* = 0.008, figure 4).

### Correlations Between Texture Features and Clinical and Serological Parameters

In the correlation analysis with mortality in days, the correlations were identified with texture features derived from the co-occurrence matrix group.

**Figure 5.**
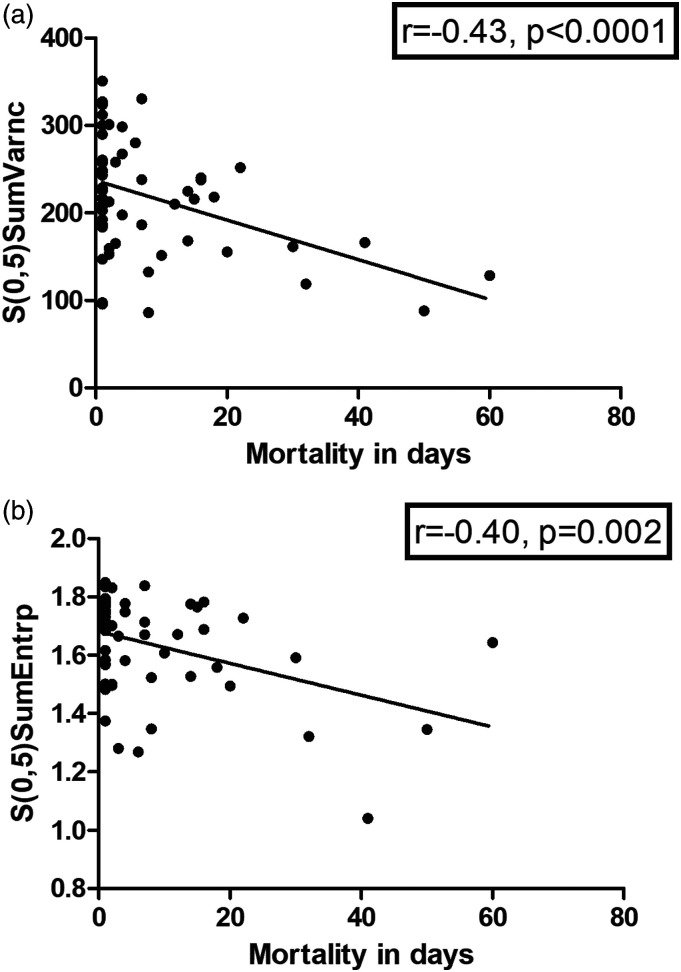
Correlation analysis between texture features of thrombotic emboli and mortality (days). A. S(0,5)SumVarnc (*r* = −0.43, *P* < 0.001). B. S(0,5)SumEntrp (*r* = −0.40, *P* = 0.002).

In detail, the following texture features correlated with mortality in days: S(0,5)SumVarnc (*r* = −0.43, *P* <0.001) (figure 5A), S(0,5)SumEntrp (*r* = −0.40, *P* = 0.002) (figure 5B), S(4,4)Correlat (*r* = −0.39, *P* = 0.003), S(5,5)Correlat (*r* = −0.38, *P* = 0.004), S(4,-4)SumEntrp (*r* = −0.38, *P* = 0.004). In Cox regression analysis including Wells score, GENEVA score, age and heart rate, these texture features contributed to the survival model. An example for S(0,5)SumVarnc is shown in Table 7.

**Table 7.** Cox Regression Analysis for Lethal Outcome in Pulmonary Artery Embolism.

Parameter	HR	95% CI	*P*-value
Wells score	0.79	0.59–1.07	0.12
Geneva score	1.13	0.96–1.34	0.15
Age at embolism	0.99	0.98–1.01	0.43
Heart rate	1.00	0.99–1.01	0.68
S(0,5)SumVarnc	1.01	1.00–1.01	0.01

Abbreviation: CI = Confidence interval, HR = hazard ratio.

**Figure 6.**
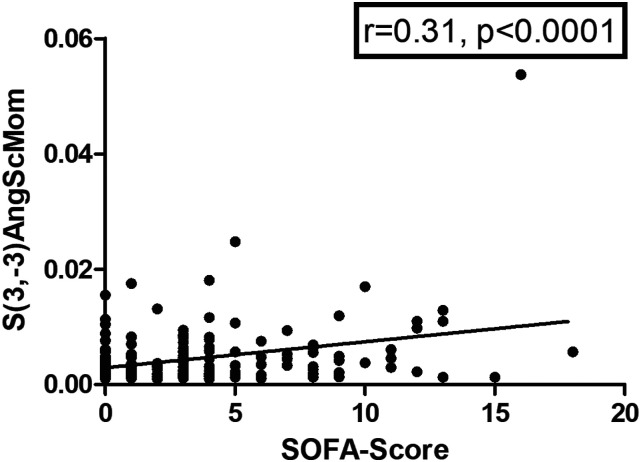
Correlation between S(3,-3)AngScMom and sepsis-related organ failure assessment score (SOFA)-score (*r* = 0.31, *P* < 0.001).

There were no associations between texture features and heart rate or blood pressure. S(3,-3)AngScMom derived from the co-occurrence matrix group showed a statistically significant correlation with SOFA-score (*r* = 0.31, *P* < 0.001) (figure 6). There were no correlations with the other investigated scores.

**Figure 7.**
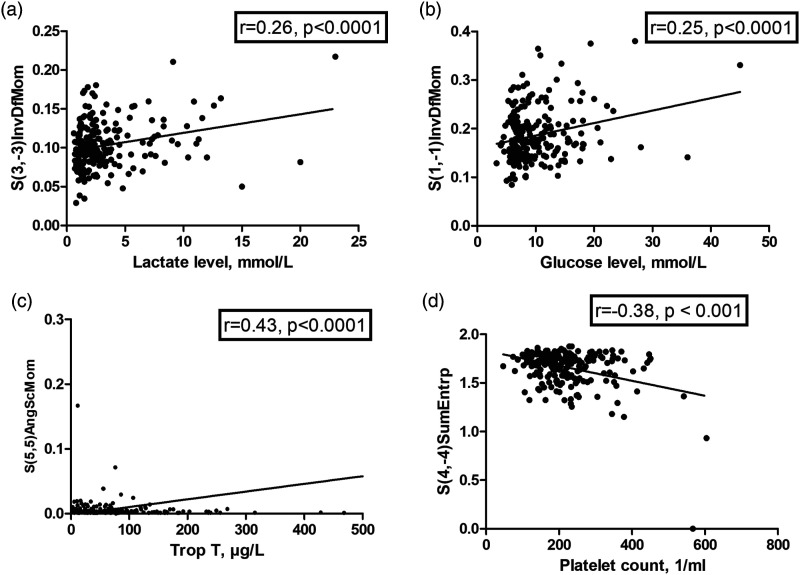
Correlation between several texture analysis features and serological parameters in patients with acute pulmonary embolism. A. Venous lactate levels and S(3,-3)InvDfMom (*r* = 0.26, *P* < 0.001). B. Venous glucose levels and S(1,-1)InvDfMom (*r* = 0.25, *P* < 0.001). C. Troponin T and S(5,5)AngScmom (*r* = 0.43, *P* < 0.001). D. Platelet count and S(4,-4)SumEntrp (*r* = −0.38, *P* < 0.001).

Several texture features correlated with venous lactate levels, for example, S(2,-2)InvDfMom (*r* = 0.25, *P* < 0.001) and S(3,-3)InvDfMom (*r* = 0.26, *P* < 0.001) (Figure 7A).

Texture features correlated slightly with venous glucose levels: S(1,-1)InvDfMom (*r* = 0.25, *P* < 0.001) (Figure 7B), and S(3,0)InvDfMom (*r* = 0.24, *P* < 0.001). S(5,5)AngScMom correlated with troponin T (*r* = 0.43, *P* < 0.001) (Figure 7C). All identified texture features were of the co-occurrence matrix group.

There were no correlations between texture analysis parameters and D-Dimer levels, BNP, and venous pH.

Several texture features were correlated with platelet count, strongest correlations were found for S(4,-4)SumEntrp (*r* = −0.38, *P* < 0.001, Figure 7D), and for S(3,-3)AngScMom (*r* = 0.34, *P* < 0.001). Also, several texture features were correlated with the international normalized ratio (INR), the highest correlation coefficient showed S(0,2)SumAverg (*r* = 0.21, *P* = 0.002).

For partial thromboplastin time (pTT), WavEnLL_s-3 showed an inverse correlation (*r* = −0.18, *P* = 0.013). Fibrinogen level was associated with S(0,3)SumVarnc (*r* = −0.36, *P* = 0.049).

Finally, several texture features correlated with hematocrit, for example, S(0,5)AngScMom (*r* = −0.26, *P* < 0.001) and S(0,5)SumEntrp (*r* = 0.25, *P* < 0.001).

Moreover, several associations were identified between texture features and hemoglobin: S(5,-5)Entropy (*r* = 0.25, *P* < 0.001) and S(0,5)AngScMom (*r* = 0.−26, *P* < 0.001).

## Discussion

The present study sought to establish texture analysis assessment of pulmonary emboli and to elucidate, whether texture features are associated with mortality and clinical parameters in patients with acute PE. This present analysis tried to utilize a possible novel embolism characterization in acute PE based upon CT images.

As presented, there are several statistically significant associations between CT texture features with mortality. With this analysis approach, a novel method can provide prognostic relevant factors in this possible life-threatening disease. Most promising texture features of the present analysis were derived from the co-occurrence matrix group. These matrices determine how often a pixel of intensity finds itself within a certain relationship to another pixel of intensity and therefore quantified the homogeneity or heterogeneity of the analyzed CT image.^24^

The topic of texture analysis is an emergent field of research with extensive studies in several diseases, predominantly in oncologic imaging.^9-11^ It has been shown that several texture features derived from CT as well as MRI images reflect distinctive histopathologic characteristics of tumors on a microstructure level.^23-26^ So far, T1- and T2-weighted texture features to reflect cellularity and nucleic areas in thyroid tumors.^23^ Furthermore, CT texture features were associated with hypoxia-related immunohistochemical features in head and neck cancer.^25^ Finally, CT texture analysis was even able to predict the complex immune milieu in lung cancer patients to guide treatment.^27^

Therefore, the principal hypothesis of this present work was that texture analysis can also reflect relevant structural differences of the embolus of the pulmonary arteries.

Risk stratification is crucial for patients with acute PE. In the clinical guidelines, an important factor for massive or critical course is hypotension with systolic blood pressure <90 mmHg.^3,28-30^ However, the absence of hemodynamic instability does not exclude beginning with a possibly progressing right ventricular dysfunction.^30^ A clinical evaluation includes the Geneva and Wells score for a first assessment of the patients.^30^ For laboratory biomarkers, elevated troponin concentrations are associated with a worse prognosis.^30^ Elevated B-type natriuretic peptide indicates right ventricular overload and is also associated with a worse prognosis. In a similar fashion, elevated C-reactive protein levels were associated with right ventricular dysfunction, which could be used as a predictor for prognosis.^31^

In a recent study, the importance of serum albumin level for short-term mortality was identified in a retrospective study of 269 patients.^32^ The authors proposed possible explanations that low level of serum albumin may result in deficiency of anti-oxidant function in acute PE patients.^32^

The imaging modalities echocardiography and CT can provide information regarding right ventricular dysfunction but other reliable prognostic factors are still lacking.^3,30^ It is a known fact that the sole embolus localization divided into central or peripheral is not a significant predictor of mortality.^32^ Moreover, total clot burden assessed by CT obstruction scores are not a sufficient method for prognosis prediction and were not translated into clinical routine.^16,33^ That is why there is need for new methods to characterize the embolus by CT imaging.

The present preliminary results suggest that texture analysis could routinely be used to provide prognostic factors for clinical care.

In vitro analyses investigated different clot compositions of patients with acute PE.^34,35^ The clot fibrin fibers of patients with fatal PE were significantly lower in diameter compared with the healthy control group.^33^ Moreover, denser fibrin networks were associated with intermediate risk PE compared with those with low-risk PE.^34^ It would be very interesting to compare these in vitro analyses with the investigated CT texture features to better characterize clot composition in patients with PE and to assess, which microstructural features of clot composition are reflected by texture features. This can also be interpreted by the fact that the localization of the embolus has an influence on the texture features. Presumably, the different clot characteristics composed of thicker fibers with increased susceptibility, which was shown for central PE compared with peripheral PE might be one reason for these differences.^35^

Extensive research was undertaken to assess possible differences of the thrombi in acute ischemic stroke.^12,13^ As such, significant differences were identified between thrombi due to cardioembolic stroke with those due to other stroke causes.^36^ Moreover, thrombus CT density parameters were associated with thrombus composition defined by fibrin/platelet fractions and red blood cell counts.^36^ In a similar way, the present results should reflect distinctive differences of emboli composition in patients with ischemic stroke.

Yet, compared with ischemic stroke, there is still lack of direct analyses of the emboli in acute PE, as surgical embolectomy is rarely performed compared with interventional mechanical thrombectomy in patients with ischemic stroke.

Beyond that, no interventional pulmonary thrombectomy was performed for the investigated patients in the present analysis and therefore the present results cannot be translated for those patients. Hypothetically, texture analysis could also aid in treatment planning of pulmonary thrombectomy in a predictive manner. Yet, further studies are needed to elucidate this interesting field for texture analysis.

This is especially of interest, as to date only a combination of right ventricular dysfunction on an echocardiogram or CT with a positive cardiac troponin test was tested as a guide for early therapeutic decisions (anticoagulation plus reperfusion treatment vs anticoagulation alone) in a large randomized controlled trial of acute PE patients.^37^ Imaging guided treatment decisions could be performed by quantitative CT texture features.

The present analysis has some inherent limitations. First, it is a retrospective study with possible bias. To reduce possible bias, texture analysis was performed blinded to the clinical results. Second, the patient sample is relatively small due to the single center design. Furthermore, selection bias might have influenced the present results. Moreover, it should be acknowledged that the present patient sample has a relatively high mortality rate. Therefore, the identified associations might not transferrable to patient samples with a lower mortality rate. Moreover, no interventional treatment was performed, which could limit the translation of the present results to patients undergoing interventional treatment. Third, texture analysis still lacks standardization. There is a need to employ the investigated texture features in other patient samples scanned with different CT scanner to test for external validation of the present results. This is needed before the presented results can be translated into clinical care. Fourth, the texture analysis measurement only included the most representative embolus of the patients and did not include every embolus of the patients. A measurement of all visible emboli of the patient might result in different results. However, this approach would not be suitable for clinical routine. Fifth, we could not perform correlation analysis for some prognostic relevant parameters, such as C-reactive protein or serum albumin due to lack of data.

## Conclusions

Several texture features of pulmonary emboli derived from CTPA were associated with mortality, need for ICU admission, clinical and serological parameters in patients with acute PE. This highlights the possible clinical benefit of those novel imaging biomarkers.
